# Resistance to tuberculin skin test/interferon-gamma release assay conversion among highly TB exposed, HIV infected goldminers in South Africa

**DOI:** 10.1371/journal.pone.0318819

**Published:** 2025-05-09

**Authors:** Thobani Ntshiqa, Kavindhran Velen, Sibuse Ginindza, Lindiwe Nhlangulela, Salome Charalambous, Thomas R. Hawn, Gavin Churchyard, W. Henry Boom, Violet Chihota, Robert Wallis

**Affiliations:** 1 The Aurum Institute, Johannesburg, South Africa; 2 School of Public Health, University of the Witwatersrand, Johannesburg, South Africa; 3 Department of Medicine, Vanderbilt University, Tennessee, United States of America; 4 Department of Medicine, University of Washington, Seattle, United States of America; 5 Department of Molecular Biology and Microbiology, Division of Infectious Diseases and HIV Medicine, School of Medicine, Case Western Reserve University, Cleveland, Ohio, United States of America; 6 Division of Infectious Diseases, Department of Medicine, Vanderbilt University School of Medicine, Nashville, Tennessee, United States of America; Shandong Public Health Clinical Center: Shandong Provincial Chest Hospital, CHINA

## Abstract

**Background:**

A small proportion of goldminers in South Africa resist tuberculin skin test (TST)/interferon-gamma release assay (IGRA) conversion despite high rates of HIV and prolonged exposure to TB. We conducted a study among HIV-infected goldminers to determine the: i) proportion who resisted TST/IGRA conversion and ii) epidemiological factors associated with resistance to TST/IGRA conversion.

**Methods:**

We enrolled HIV-infected goldminers who were on antiretroviral treatment, aged 33–60 years, with ≥15 years’ service, no prior or current TB, no silicosis, and with body mass index >18.5 kg/m^2^. TST/IGRA conversion was assessed at baseline, 6 months, and 12 months using TST and QuantiFERON-TB-Gold-Plus (QFT-Plus). Miners were considered resisters if they had a zero TST response and a negative QFT-Plus at all visits. Logistic regression was used to identify epidemiological factors associated with TST/IGRA conversion resistance.

**Results:**

We enrolled 245 HIV-infected miners with median age of 48 years (interquartile-range [IQR]: 44–52 years) and median CD4 count, 506 cells/ µ L (IQR: 372–677 cells/ µ L). Overall, 98.4% (241) were males and 99.2% (243) were Black/African with a median time of 24 years (IQR: 18–29 years) in the workforce. Of those completing all follow-ups, 24.3% (50/206) resisted TST/IGRA conversion. Miners who had a history of taking isoniazid preventive therapy (IPT) (adjusted odds ratio (aOR) 2.34; 95% confidence interval (CI): 1.14–4.80; p = 0.020) were more likely to resist TST/IGRA conversion. However, those from Mozambique (aOR 0.16; 95% CI: 0.04–0.71; p = 0.016) and those who had a CD4 count ≥500 cells/ µ L (aOR 0.46; 95% CI: 0.23–0.92; p = 0.028) were less likely to resist TST/IGRA conversion.

**Conclusion:**

Similar to previous longitudinal cohort studies, we found a small proportion of HIV-infected goldminers who resisted TST/IGRA conversion. This was positively associated with prior IPT, but negatively associated with lower CD4 count and being from Mozambique. However, mechanisms underlying TST/IGRA conversion resistance are not well understood.

## Introduction

Tuberculosis (TB), caused by *Mycobacterium tuberculosis* (*Mtb*), is a second leading cause of mortality worldwide from a single infectious agent [1]. Although the true global burden of *Mtb* infection is not well known, it was estimated around 23% in 2014 [2]. In Sub-Saharan Africa, the TB epidemic is further exacerbated by HIV infection. Goldmines are considered to be one of the major transmission hotspots for TB with an estimated incidence of TB disease of 3% [3]. In addition, the annual risk for *Mtb* infection in goldmines was estimated at 20% in a recent mathematical modeling study [3] Compared to general population, this estimate is five times higher.. TB prevalence was estimated at 2,500–3,000 cases per 100,000 within the workforce in South African mines in 2017 [4,5]. In the same period, TB incidence was estimated to be 10 times higher than the WHO threshold for a health emergency, and three times higher than the incidence in general population [4,5].

Prolonged, repeated exposure and infectiousness of TB index patient are known to increase the risk of becoming infected with *Mtb* [6]. In the absence of TB preventive therapy post infection, approximately 5–10% of the immunocompetent and ≥ 40% of immunocompromised persons may progress to clinical disease in their lifetime or in the first several years, respectively [7–9]. Not all persons exposed to *Mtb* become infected. This suggests that some individuals may either resist *Mtb* infection or rapidly clear it through innate immune responses [7,10–12], as evidenced by a persistently negative tuberculin skin test (TST) or interferon-gamma release assay (IGRA) despite prolonged exposure to TB in longitudinal cohorts of household contacts of persons with active pulmonary TB disease in Uganda [13–15], India [16], Indonesia [17], and South African goldmines [18–21]. In addition, a recent mathematical modelling study has estimated a 93% likelihood of having a resister phenotype in goldminers who are persistently uninfected with *Mtb* despite repeated exposure to infectious TB cases over extended period of time [6].

Earlier evidence from studies conducted in goldmines suggests that the mining population could provide a unique opportunity to identify individuals that have been exposed to TB repeatedly over decades yet remain uninfected, to gain insight into biomarkers and mechanisms of resistance to *Mtb* infection [20]. It is hypothesised that the true proportion of *Mtb* resisters among HIV-infected non anergic people may be high since HIV increases the risk of progression to active TB disease [6,20]. In addition, a monocyte transcriptional response study conducted among household contact in Uganda found that HIV-infected *Mtb* resisters had a different gene expression profile in response to *Mtb* infection compared with *Mtb* infected and HIV negative *Mtb* resisters [22]. However, it may be important to study this in different settings and populations as the effect of HIV on an individual’s ability to resist developing *Mtb* infection would be particularly important given its prominence in many high-burden TB settings.

Studies previously conducted in the mines, did not determine risk factors for *Mtb* infection in HIV-infected individuals [19]. Most recently, a small proportion of HIV negative goldminers who appeared to resist *Mtb* infection were identified in South Africa, however no clear distinguishing epidemiological characteristics were identified [20]. Similarly, no epidemiological factors were associated with the resister phenotype in household contacts [16,23,24]. Therefore, studying HIV-infected individuals to determine epidemiological factors associated with remaining *Mtb* uninfected despite being highly exposed remains a priority. We conducted a study among HIV-infected goldminers to determine: i) the proportion who were resistant to *Mtb* infection and ii) epidemiological factors associated with resistance to *Mtb* infection.

## Materials and methods

### Study setting and population

The study was conducted in three South African goldmines: two in Orkney, North-West Province from 10^th^ of July 2017–31^st^ of September 2019 and one in Carletonville, Gauteng Province from 17^th^ of October 2018–31^st^ of September 2019. However, twelve-month follow-up visits were stopped on the 23^rd^ of March 2020 in all sites due to COVID-19 lockdown. The target population was HIV-infected goldminers on antiretroviral treatment (ART) for ≥ 3 months, who worked for ≥ 15 years in goldmines but with no prior history of TB disease nor evidence of silicosis [20]. In addition, study participants were included in the study only if they were aged 33–60 years and had body mass index (BMI) >18.5 kg/m^2^ [20]. This eligibility criteria were designed to maximise recruiting miners that had a resister phenotype.

### Study design and procedures – main study

In a cohort study, herein described as the main study, we enrolled HIV-infected miners who were on ART. However, we did not collect data on ART regimen and history of default or treatment failure. To identify individuals who had high cumulative exposure to TB and yet remain resistant to *Mtb* infection, we screened potential participants for eligibility in two phases as described below. High cumulative exposure in our context was based on proxies of age, and years in the workforce; no TB disease; or not being at higher risk of TB disease. This is also based on the assumption of the high force of infection reported in Vynnycky et., al [3]. Cumulative exposure also refers to individuals who have worked in mining settings of high force of *Mtb* infection coupled with exposure to silica dust underground.

### Pre-screening phase

Goldminers, attending wellness clinics within the mining hospital and/or medical stations for their routine HIV care between July 2017 and September 2019, were invited to join the study. During pre-screening, we ascertained age and years working in the mining industry while miners were waiting to see a clinician for their routine HIV care. Individuals identified as having worked for prolonged periods in the mining industry (at least 15 years) and who were at least 33 years of age were consented [20].

### Full screening phase

Following an informed consenting process, a full screen was conducted among all those who met the pre-screening requirements and individuals who did not meet an earlier stage were excluded. A questionnaire was administered to collect information on medical history, including prior and current treatment for TB disease and *Mtb* infection. In addition, a symptom screen for active TB disease was performed using the WHO screening tool comprising 1) current cough of any duration; 2) fever; 3) night sweats; or 4) unintended weight loss. The most recent chest radiograph (≤3 months at a time of recruitment by study team) taken as part of routine annual examination at the Occupational Health Centre were reviewed for evidence of old or active TB and silicosis. Miners were enrolled into the study if 1) they did not report symptoms suggestive of TB; 2) no prior or current history of treatment for active TB disease was documented; 3) no evidence of silicosis documented; 4) BMI > 18.5 kg/m^2^; and 5) no serious or chronic medical conditions causing severe immunodeficiency. Miners also had to be on ART for ≥ 3 months with a CD4 ≥ 200 cells/ µ L but not current taking treatment for cancer, no treatment with steroid tablets, inhalers, or injections. The following procedures were conducted after full screening was completed among all miners who met the inclusion criteria.

### Clinical and lab procedures: Evaluation of *Mtb* infection using TST and QFT-Plus

At enrolment, participants gave blood samples for QFT-Plus and had a TST placed (RT-23 in Tween-80, Statens Serum Institute, Copenhagen, Denmark, catalogue number: XI1177KL and SP18411C) by trained professional nurses and read after 48–72 hours. The TST response was measured using a digital calliper as the maximum transverse diameter of the induration expressed in millimetres. Blood samples were collected from those who met the inclusion criteria for QFT-Plus (Qiagen, Hilden Germany) by phlebotomy trained professional nurses. All participants with a negative QFT-Plus (according to manufacturer’s instruction) at enrolment, were followed up at 6 months and 12 months after baseline. In both follow-up visits QFT-Plus and TST testing were repeated.

### Study design – sub-analysis and definitions

We determined the proportion of miners with *Mtb* resistance and explored epidemiological factors associated with *Mtb* resistance using measurements from baseline, 6 months, and 12 months follow-up. *Mtb* infection status was confirmed using TST and QFT-Plus, and a questionnaire was administered to collect information on factors associated with being *Mtb* resister. QFT-Plus was considered negative if both antigen tubes (TB1 and B2) were <0.35 IU^.^ml^-l^. Miners were considered *Mtb* resisters if they had a zero TST (induration = 0mm) and negative QFT-Plus (response <0.35 IU^.^ml^-l^ on both TB1 and TB2 antigen tubes) at all three time points. Lost to follow up was defined as any miner who consented to participate or participated in this study at one point in time but was lost or could not be contacted to complete the outstanding study procedures on subsequent follow-ups.

### Study outcome

The study outcome was the proportion of participants who had a zero TST (induration = 0mm) AND negative QFT-Plus (response <0.35 IU^.^ml^-l^ on both TB1 and TB2 antigen tubes) at baseline, 6 months, and 12 months follow-up visits, herein referred to as *Mtb* resister. Participants with a positive TST or QFT at any time point were the comparator group.

### Data management

All data including participant identifiers, enrolment and follow-up questionnaires were collected using an electronic data capture system (IBM eClinical).

### Statistical methods

Demographic characteristics were described using frequencies (n) and percentages (%), and Chi-square (*χ*^2^) test or two-sided Fisher’s exact test was used to compare categorical variables. For statistical comparisons, a p ≤ 0.05 was considered significant. The univariable and multivariable logistic regression models were used to determine predictors or factors associated with a study outcome. Predictors with p < 0.20 in the univariable logistic regression model were introduced into the multivariable model. Manual forward and backward stepwise procedure were used to select variables for the multivariable model. A cut-off p < 0.05 was used to retain variables in the final multivariable model. Results were summarized using odds ratios (OR) and adjusted odds ratios (aOR) with their corresponding 95% confidence intervals (CI) and p-values. Data was analysed using Stata version 15 (StataCorp. 2017. Stata Statistical Software: Release 15. College Station, TX: StataCorp LP).

### Ethics statement

The study received ethical clearance from the University of Witwatersrand Human Research Ethics Committee (WHREC Ref: 170110), University of Washington (IRB#: STUDY00001537), North West Health Research and Ethics Committee (NW_2017RP26_755), and Gauteng Health Research and Ethics Committee (GP_201804_024). Permission was also sought from internal stakeholders within the mining companies where the study was conducted. Prior to conducting study procedures, we sought informed consent from all study participants using written informed consent and information sheet available in the commonly used local languages. An impartial witness was used to witness the verbal consent for illiterate participants. A thumb print was used to document or “sign” a verbal consent.

### Inclusivity in global research

Additional information regarding the ethical, cultural, and scientific considerations specific to inclusivity in global research is included in the Supporting Information (S1 File).

## Results

### Participants screening and enrolment

**Fig 1:**
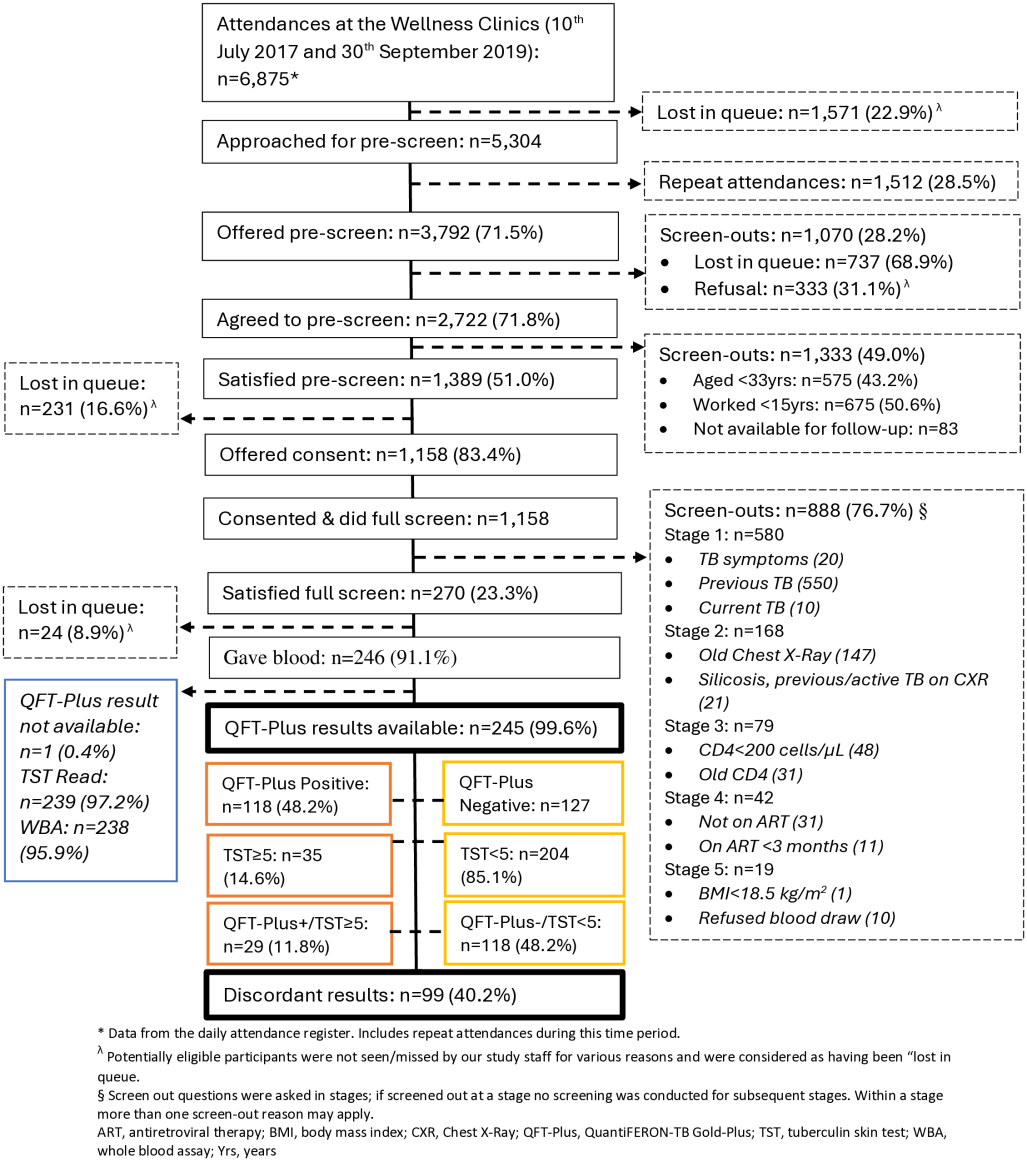
Participants flow chart at enrolment among miners attending HIV wellness clinic in South African gold mines.

Between the 10^th^ of July 2017 and 30^th^ of September 2019, a total of 6,875 visits were recorded at the wellness clinics from a mining hospital and medical stations (Fig 1). We approached 5,304 miners (77.1%), 2,722 (71.8%) were pre-screened and remainder were either lost in queue or declined to take part in the study. Of those who were pre-screened, 1,389 (51.0%) were eligible for full screening, of whom 1,158 (83.4%) consented and completed full screening (Fig 1).

**Fig 2:**
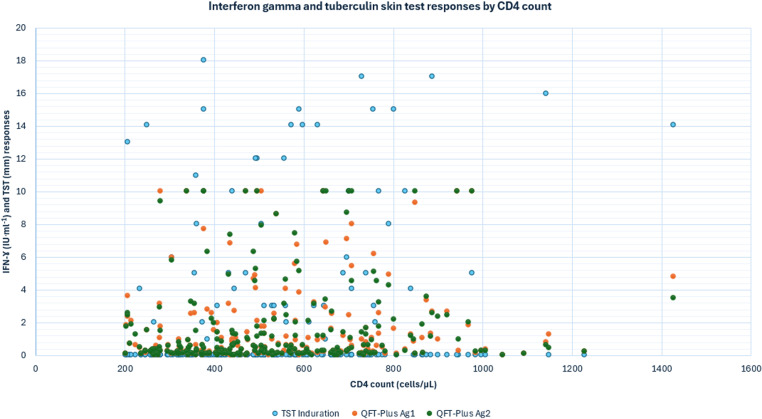
Interferon gamma response and tuberculin skin test responses by CD4 count among miners attending HIV wellness clinic in South African gold mines.

Among the consented participants who completed full screening, 270 (23.3%) met the inclusion criteria; 888 (76.7%) were screened out. Of those meeting the inclusion criteria, 246 (91.1%) were enrolled and 24 (8.9%) were either lost in queue or lost to follow up. In total, 246 participants were enrolled, all gave blood for QFT-Plus testing, and TST was placed. Of the 246 enrolled miners, 245 (99.6%) had baseline QFT-Plus results and 239 (97.2%) had TST results (Fig 1). Fig 2 shows the interferon gamma response and TST induration by CD4 count.

### Demographic characteristics of participants

In this analysis, we included 245 participants with the median age of 48 years (interquartile-range [IQR]: 44–52 years) and 98.4% (241) were male. The median time in the workforce was 24 years (IQR: 18–29 years), median CD4 count was 506 cells/ µ L (IQR: 372–677 cells/ µ L) (Table 1). Overall, 99.2% (243) were of Black/African ethnicity, 74.3% (179) had a Bacillus Calmette Guerin (BCG) vaccination scar present, 46.9% (115) were born in South Africa and half (50.6%, 124) were staying in hostels (Table 1).

**Table 1:** Baseline demographic characteristics of miners attending HIV wellness clinic in South African gold mines.

Variable (N = 245)		*Resister phenotype status		N (%)
		*Mtb* resisters – n (%)	Non-resisters – n (%)	
		50 (20.4%)	195 (79.6%)	245 (100.0%)
**Employment duration**				
	Median (IQR)	23.5 (17–29)	24 (19–29)	24 (18–29)
**Years underground**				
	Median (IQR)	21 (16–29)	24 (18–28)	23 (17–28)
**Years ART**				
	Median (IQR)	4 (2–9)	4 (3–8)	4 (2–8)
**CD4 Count**				
	Median (IQR)	418 (332–553)	530 (377–704)	506 (372–677)
**Age, years**				
	Median (IQR)	48 (44–51)	48 (44–52)	48 (44–52)
**Age group, years**				
	<45	15 (21.1%)	56 (78.9%)	71 (29.0%)
	45-49	17 (23.3%)	56 (76.7%)	73 (29.8%)
	≥50	18 (17.8%)	83 (82.2%)	101 (41.2%)
**Gender**				
	Female	2 (50.0%)	2 (50.0%)	4 (1.6%)
	Male	48 (19.9%)	193 (80.1%)	241 (98.4%)
**Ethnicity**				
	Other	0 (0.0%)	2 (100.0%)	2 (0.8%)
	Black/African	50 (20.6%)	193 (79.4%)	243 (99.2%)
**Country of origin**				
	Other	7 (30.4%)	16 (69.6%)	23 (9.4%)
	Mozambique	4 (9.3%)	39 (90.7%)	43 (17.6%)
	Lesotho	9 (14.1%)	55 (85.9%)	64 (26.1%)
	South Africa	30 (26.1%)	85 (73.9%)	115 (46.9%)
**Marital status**				
	Not married	10 (26.3%)	28 (73.7%)	38 (15.5%)
	Married	40 (19.3%)	167 (80.7%)	207 (84.5%)
**Occupation**				
	Skilled	9 (13.9%)	56 (86.2%)	65 (26.5%)
	Unskilled	41 (22.8%)	139 (77.2%)	180 (73.5%)
**Live in hostel**				
	No	29 (24.0%)	92 (76.0%)	121 (49.4%)
	Yes	21 (16.9%)	103 (83.1%)	124 (50.6%)
**Sleeping arrangement**				
	Alone	16 (20.5%)	62 (79.5%)	78 (31.8%)
	1 person	29 (19.7%)	118 (80.3%)	147 (60.0%)
	> 1 person	5 (25.0%)	15 (75.0%)	20 (8.2%)
**BCG scar**				
	No	14 (22.6%)	48 (77.4%)	62 (25.7%)
	Yes	35 (19.6%)	144 (80.4%)	179 (74.3%)
**BMI**				
	≤24	17 (18.5%)	75 (81.5%)	92 (37.6%)
	25-29	22 (20.8%)	84 (79.2%)	106 (43.3%)
	≥30	11 (23.4%)	36 (76.6%)	47 (19.2%)
**Currently working underground**				
	No	7 (35.0%)	13 (65.0%)	20 (8.2%)
	Yes	43 (19.1%)	182 (80.9%)	225 (91.8%)
**CD4 Count**				
	<500	33 (27.1%)	89 (72.9%)	122 (49.8%)
	≥500	17 (13.8%)	106 (86.2%)	123 (50.2%)
**Years on ART**				
	<5	28 (22.2%)	98 (77.8%)	126 (51.4%)
	≥5	22 (18.5%)	97 (81.5%)	119 (48.6%)
**IPT ever**				
	No	27 (15.6%)	146 (84.4%)	173 (70.6%)
	Yes	23 (31.9%)	49 (68.1%)	72 (29.4%)
**Currently on IPT**				
	No	44 (19.4%)	183 (80.6%)	227 (92.7%)
	Yes	6 (33.3%)	12 (66.7%)	18 (7.3%)

ART, antiretroviral therapy; BCG, Bacillus Calmette Guerin; BMI, body mass index; CD4, Clusters of differentiation 4; IPT, isoniazid preventive therapy; IQR, interquartile-range; QFT-Plus, QuantiFERON-TB Gold-Plus; TST, tuberculin skin test

*Mtb resister phenotype, defined as having a QFT-Plus Negative/TST = 0mm results at baseline, 6 & 12 months follow up.

### *Mtb* resister phenotype

**Fig 3:**
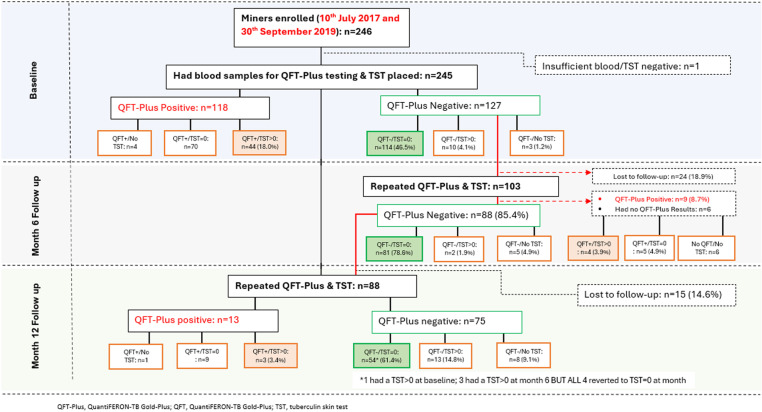
Participants flow chart showing phenotype stability at follow-up visit among miners attending HIV wellness clinic in South African gold mines.

Of those who had QFT-Plus results at baseline, 6 months follow-up, and 12 months follow-up, 52.8% (127/245), 85.4% (88/103), and 85.2% (75/88) were QFT-Plus negative, respectively (Fig 3). On the other hand, the proportion of those with a zero TST response and a negative QFT-Plus result was 46.5% (114/245) at baseline, 78.6% (81/103) at 6 months follow-up, and 61.4% (54/75) at 12 months follow-up. Of the 54 participants that had a zero TST response and a negative QFT-Plus result at 12 months follow-up, 1 had TST > 0mm at baseline while 3 had TST > 0mm at 6 months follow-up. As a results, the 4 participants were not considered as *Mtb* resister in the final analysis. **Therefore, the overall proportion of miners who met the definition for *Mtb* resistance was 24.3% (50/206).**

### Epidemiological factors associated with *Mtb* resistance

On univariable analysis, being *Mtb* resister was positively associated with a prior history of taking isoniazid preventive therapy (IPT) (OR 2.54; 95% CI: 1.33–4.83; p = 0.005), but negatively associated with being from Mozambique (OR 0.23; 95% CI: 0.06–0.91; p = 0.036) and having a CD4 count of ≥ 500 cells/ µ L (OR 0.43; 95% CI: 0.23–0.83; p = 0.011) (Table 2). On multivariable analysis, miners who had a prior history of taking IPT (aOR 2.34; 95% CI: 1.14–4.80; p = 0.020) remained two times more likely to resist *Mtb* infection. On the other hand, miners from Mozambique (aOR 0.16; 95% CI: 0.04–0.71; p = 0.016) and those who had a CD4 count ≥500 cells/ µ L (aOR 0.46; 95% CI: 0.23–0.92; p = 0.028) remained less likely to resist *Mtb* infection (Table 2).

**Table 2:** Factors associated with Mtb resistance, defined as having a zero TST response and a negative QFT-Plus result at the end of follow-up period, among miners attending HIV wellness clinic in South African gold mines.

Variable (N = 245)		*Mtb* resisters		Univariable analysis			Multivariable analysis		
		N	%	Crude OR	95% CI	p-value	Adjusted OR	95% CI	p-value
Age group, years									
	*<45*	15	21.1	1	Reference	Reference	1	Reference	Reference
	*45-49*	17	23.3	1.13	0.52 - 2.49	0.755	0.92	0.39 - 2.16	0.842
	*≥50*	18	17.8	0.81	0.38 - 1.74	0.588	0.68	0.29 - 1.57	0.362
Gender									
	*Female*	2	50.0	1	Reference	Reference	1	Reference	Reference
	*Male*	48	19.9	0.25	0.03 - 1.81	0.170	0.41	0.04 - 3.79	0.432
Country									
	*Other*	7	30.4	1	Reference	Reference	1	Reference	Reference
	*Mozambique*	4	9.3	0.23	0.06 - 0.91	0.036	0.16	0.04 - 0.71	0.016
	*Lesotho*	9	14.1	0.37	0.12 - 1.16	0.089	0.36	0.11 - 1.20	0.097
	*South Africa*	30	26.1	0.81	0.30 - 2.15	0.668	0.56	0.19 - 1.65	0.296
Marital status									
	*Not married*	10	26.3	1	Reference	Reference	–	–	–
	*Married*	40	19.3	0.67	0.30 - 1.49	0.328	–	–	–
Occupation									
	*Skilled*	9	13.9	1	Reference	Reference	1	Reference	Reference
	*Unskilled*	41	22.8	1.84	0.84 - 4.03	0.130	1.64	0.71 - 3.82	0.249
Live in hostel									
	*No*	29	24.0	1	Reference	Reference	1	Reference	Reference
	*Yes*	21	16.9	0.65	0.35 - 1.21	0.174	0.74	0.36 - 1.52	0.414
Sleeping arrangement									
	*Alone*	16	20.5	1	Reference	Reference	–	–	–
	*1 person*	29	19.7	0.95	0.48 - 1.89	0.889	–	–	–
	*> 1 person*	5	25.0	1.29	0.41 - 4.09	0.663	–	–	–
BCG scar									
	*No*	14	22.6	1	Reference	Reference	–	–	–
	*Yes*	35	19.6	0.83	0.41 - 1.68	0.610	–	–	–
BMI									
	*≤24*	17	18.5	1	Reference	Reference	–	–	–
	*25-29*	22	20.8	1.16	0.57 - 2.34	0.688	–	–	–
	*≥30*	11	23.4	1.35	0.57 - 3.17	0.494	–	–	–
Currently working underground									
	*No*	7	35.0	1	Reference	Reference	1	Reference	Reference
	*Yes*	43	19.1	0.44	0.17 - 1.17	0.098	0.41	0.13 - 1.27	0.121
CD4 Count									
	*<500*	33	27.1	1	Reference	Reference	1	Reference	Reference
	*≥500*	17	13.8	0.43	0.23 - 0.83	0.011	0.46	0.23 - 0.92	0.028
Years on ART									
	*<5*	28	22.2	1	Reference	Reference	–	–	–
	*≥5*	22	18.5	0.79	0.42 - 1.48	0.469	–	–	–
IPT ever									
	*No*	27	15.6	1	Reference	Reference	1	Reference	Reference
	*Yes*	23	31.9	2.54	1.33 - 4.83	0.005	2.34	1.14 - 4.80	0.020
Currently on IPT									
	*No*	44	19.4	1	Reference	Reference	1	Reference	Reference
	*Yes*	6	33.3	2.08	0.74 - 5.85	0.165	1.86	0.59 - 5.86	0.292

ART, antiretroviral therapy; BCG, Bacillus Calmette Guerin; BMI, body mass index; CD4, Clusters of differentiation 4; IPT, isoniazid preventive therapy; IQR, interquartile-range; QFT-Plus, QuantiFERON-TB Gold-Plus; TST, tuberculin skin test.

## Discussion

We found a small proportion of HIV-infected goldminers on ART who were *Mtb* resisters. Prior history of taking IPT was associated with a greater likelihood of having *Mtb* resister phenotype. In contrast, coming from Mozambique and having a CD4 count ≥500 cells/ µ L were associated with a lower likelihood of having a resister phenotype. Findings from this study contributes to existing scientific literature which suggests that individuals with *Mtb* resister phenotype do exist irrespective of HIV status, intensity and duration of exposure to TB [20].

The proportion of *Mtb* resister phenotype reported in in this study (24.3%) was within a similar range (0–35%) reported in previous study [18,20]. However, it was almost two times higher than the proportion (10.6%) reported among HIV-negative goldminers from the similar workforce [20]. The *Mtb* resister proportion reported in this study was also much higher than the *Mtb* resister proportion reported in several household contact cohorts with different force of infection profiles [13,16,24]. For instance, a multi-country study conducted among household contacts of multi-drug resistant TB cases in Botswana, Brazil, Haiti, India, Kenya, Peru, South Africa, and Thailand, reported an *Mtb* resisters proportion of 9.9% [24]. Household contact studies conducted in Uganda (11.7%) and India (6.5%), reported even lower proportion of resisters [13,16]. However, some of these studies were cross-sectional studies and did not analyse *Mtb* resister phenotypes by HIV status with some only using TST to determine *Mtb* infection status and varying TST cut-offs. It therefore remains unclear how much of a role did the HIV status, CD4 count and/or anergy play in the performance of QFT-Plus and TST in these studies. Previous studies conducted in HIV-infected cohorts found that the performance of TST and IGRA can be affected by immune status of a participant, resulting in underestimation of *Mtb* infection in this population [25–30]. Specifically, those with lower CD4 count and anergy are often unable to mount adequate immune response [31–34], with notable differences in median in CD4 count between groups [26,34]. This may result in misclassification of HIV-infected participants who are anergic as having a resister phenotype. Since we did not conduct any test for anergy, it remains unclear how much of a role HIV status and CD4 count play in the performance of QFT-Plus and TST.

In household contact studies, contacts had a well-define case source with a more precise measure of intensity and duration of exposure [3]. In contrast, age and period worked in goldmines were used as a proxy for prolonged and repeated exposure to *Mtb* in our study and we did not collect data on TB contact history*.* This was informed by a modelling study which suggested a greater likelihood of having a resister phenotype in miners who test negative on IGRA and TST despite working in goldmines for extended period [6]. However, it remains unclear whether the negative QFT-Plus and/or TST in this study was due to non-exposure to *Mtb* bacilli or having *Mtb* resister phenotype. It also remains unclear whether the observed QFT-Plus and/or TST conversions, at 6- and 12-month follow-ups, may be attributed to most recent exposure rather than a prolonged exposure. In addition, a validated exposure risk score capturing cumulative exposure through indicators of exposure duration in mines does not exist. Further work is needed to determine and validate exposure risk scores for *Mtb* infection in mines.

Miners from Mozambique were less likely to have *Mtb* resister phenotype and this association was statistically significant. The association between *Mtb* resister phenotype and geographic location such as country of origin has never been reported in previous studies. However, it may suggest that settings with higher force of infection profiles, as demonstrated in previous studies [13,16,24], may have a lower proportion of *Mtb* resister phenotype.

Lastly, this study found that miners with a prior history of IPT were two times more likely to have *Mtb* resister phenotype. IPT is widely used as a prophylaxis for *Mtb* infection [35–37]. However, studies in diverse settings have reported TST or QFT-Plus reversion after IPT, particularly in persons with recent conversion and small reaction diameters [38,39]. Therefore, the observed association between *Mtb* resistance and taking IPT may be indicative of treatment efficacy rather than true resistance to *Mtb* infection.

Similar to previous studies, we did not find plausible epidemiological characteristics associated with *Mtb* resister phenotype. We hypothesize that *Mtb* resister phenotype can be explained by immunological and cellular mechanisms underlying the clinical phenomenon of resistance to *Mtb* infection. Therefore, further studies comparing cellular responses, innate immune responses and gene expression profiles are needed to provide some insight.

### Study limitations

Our study had some limitations. For instance, while it was mandatory for mines to conduct baseline and annual TB and silicosis surveillance through radiography screening, miners could still seek care outside the mining healthcare system. It is therefore possible that a very small proportion of miners who accessed care elsewhere (private and public clinics) were missed by our study team during recruitment and enrolment process. In addition, *Mtb* infection and *Mtb* resister phenotype were defined based on indirect measure of adaptive immune response to *Mtb* infection, similar to HIV negative cohort [20]. A negative TST, IGRA or non-conversion of these test may be due to inadequate exposure, involvement of innate immunity or other adaptive immune mechanisms other than interferon-gamma response [20].

Further work is needed to determine cellular immune functions associated with apparent resistance to *Mtb* infection. Potential cellular mechanisms underlying the clinical phenomenon of resistance to *Mtb* infection despite high exposure, were identified in household contacts in Uganda and among HIV negative gold miners. Having *Mtb* resister phenotype was associated with novel chromosomal loci in a genome scan study among household contacts in Uganda [15]. Further work is needed to determine genetic factors that may potentially play a key role in *Mtb* infection in this population group. Furthermore, individuals with *Mtb* resister phenotype had a distinct gene expression profiles in monocytes in a gene expression study from distinct Ugandan and South African cohorts [40,41]. Similar analysis on gene expression profiles in monocytes is planned in this population group to provide further insight.

## Conclusions

We found a small proportion of HIV-infected goldminers who had a resister phenotype. However, we did not find plausible epidemiological characteristics associated with *Mtb* resister phenotype. Our results are consistent with the study among HIV negative miners but not similar to other *Mtb* resister studies conducted in household contacts.

## Supporting information

S1 File Inclusivity in global research. (DOCX)
